# Antitumorigenic effect of insect-derived peptide poecilocorisin-1 in human skin cancer cells through regulation of Sp1 transcription factor

**DOI:** 10.1038/s41598-021-97581-0

**Published:** 2021-09-16

**Authors:** Ra Ham Lee, Jae-Don Oh, Jae Sam Hwang, Hak-Kyo Lee, Donghyun Shin

**Affiliations:** 1 Department of Animal Biotechnology, Jeonbuk National University, Jeonju, 54896 Republic of Korea; 2 Department of Agricultural Biology, National Institute of Agricultural Sciences, Rural Development Administration, Wanju, 55365 Republic of Korea; 3 The Animal Molecular Genetics and Breeding Center, Jeonbuk National University, Jeonju, 54896 Republic of Korea; 4 Department of Agricultural Convergence Technology, Jeonbuk National University, Jeonju, 54896 Republic of Korea

**Keywords:** Cancer therapy, Skin cancer, Cell death

## Abstract

Malignant melanoma is highly resistant to conventional treatments and is one of the most aggressive types of skin cancers. Conventional cancer treatments are limited due to drug resistance, tumor selectivity, and solubility. Therefore, new treatments with fewer side effects and excellent effects should be developed. In previous studies, we have analyzed antimicrobial peptides (AMPs), which showed antibacterial and anti-inflammatory effects in insects, and some AMPs also exhibited anticancer efficacy. Anticancer peptides (ACPs) are known to have fewer side effects and high anticancer efficacy. In this study, the insect-derived peptide poecilocorisin-1 (PCC-1) did not induce toxicity in the human epithelial cell line HaCaT, but its potential as an anticancer agent was confirmed through specific effects of antiproliferation, apoptosis, and cell cycle arrest in two melanoma cell lines, SK-MEL-28 and G361. Additionally, we discovered a novel anticancer mechanism of insect-derived peptides in melanoma through the regulation of transcription factor Sp1 protein, which is overexpressed in cancer, apoptosis, and cell cycle-related proteins. Taken together, this study aims to clarify the anticancer efficacy and safety of insect-derived peptides and to present their potential as future therapeutic agents.

## Introduction

Skin cancer worldwide is the leading cause of skin diseases^1^. Among them, cutaneous melanoma is growing rapidly in the white population, and its incidence has been increasing by approximately 3–7% annually over the past decades^2^. Conventionally, studies on the development of anticancer drugs that induce cell death through chemotherapy using natural extracts have been actively conducted. Representatively, methylhonokiol has been reported to have anti-tumor effects in human oral squamous cancer cell lines, and anticancer effects using various natural materials have been reported^3–5^. Additionally, significant progress has been made in recent years in the prevention, diagnosis and treatment of some types of cancer^6^. Currently, typical cancer treatments include chemotherapy, biological, radiation, surgery, and hormonal therapy. However, the high cost and side effects of most cancer treatments are major problems^7^.

Recently, as one of the various studies on cancer treatment, treatment using peptides has attracted attention, and therapeutic peptides have emerged as a new and promising approach for the development of anticancer drugs^8,9^. Peptides, usually short linear chains (Amino acids less than 50 AA in length (AA)), are often stabilized by disulfide bonds^10^. Peptides target and arrest the cancer cell cycle^11^, induce apoptosis of cancer cells^12–16^, and increase the level of apoptosis by targeting tumor suppressor proteins^17–20^. The peptide also inhibits the growth of cancer cells by targeting transcription factors to reduce tumor volume^21–24^. Currently, studies to evaluate the properties of peptides having such potential anticancer effects are actively being conducted^25^.

In addition, cationic antimicrobial peptides (AMPs) are toxic to bacteria that are not normal mammalian cells, and induce extensive cytotoxicity against cancer cells^26^. Naturally occurring AMPs, a chemical defense mechanism against various external attacks (bacteria, protozoa, fungi, and viruses) in eukaryotic cells, is one of the first evolved and successful forms^27^. According to these characteristics, insect-derived AMPs may act as anticancer peptides (ACPs) that kill cancer cells^16,27–29^.

We screened AMPs based on transcriptome analysis of the red-striped golden stink bug (*Poecilocoris lewisi*) in a previous study, and among them, ACP with anticancer efficacy was selected^30^. The selected ACP, called poecilocorisin-1 (PCC-1), did not show significant toxicity in the human epithelial cell line HaCaT, but was toxic to skin cancer cells, and induced cell cycle arrest and apoptosis. In addition, the transcription factor specificity protein 1 (Sp1) is involved in cell cycle progression and cell death as a basic transcription factor^3,31^. Therefore, to investigate the anticancer effect of PCC-1 on the transcription factor Sp1 in human skin cancer cell lines SK-MEL-28 and G361, we evaluated the anticancer activity according to the treatment concentration. Additionally, Western blotting and real-time PCR were used to analyze several important proteins and genes related to cell growth and cell cycle. Finally, by evaluating the anticancer efficacy of the insect-derived peptide PCC-1 in human skin cancer cell lines, we intend to present its potential as an anticancer agent.

## Results

### PCC-1 inhibits the viability of malignant melanoma cells

**Figure 1:**
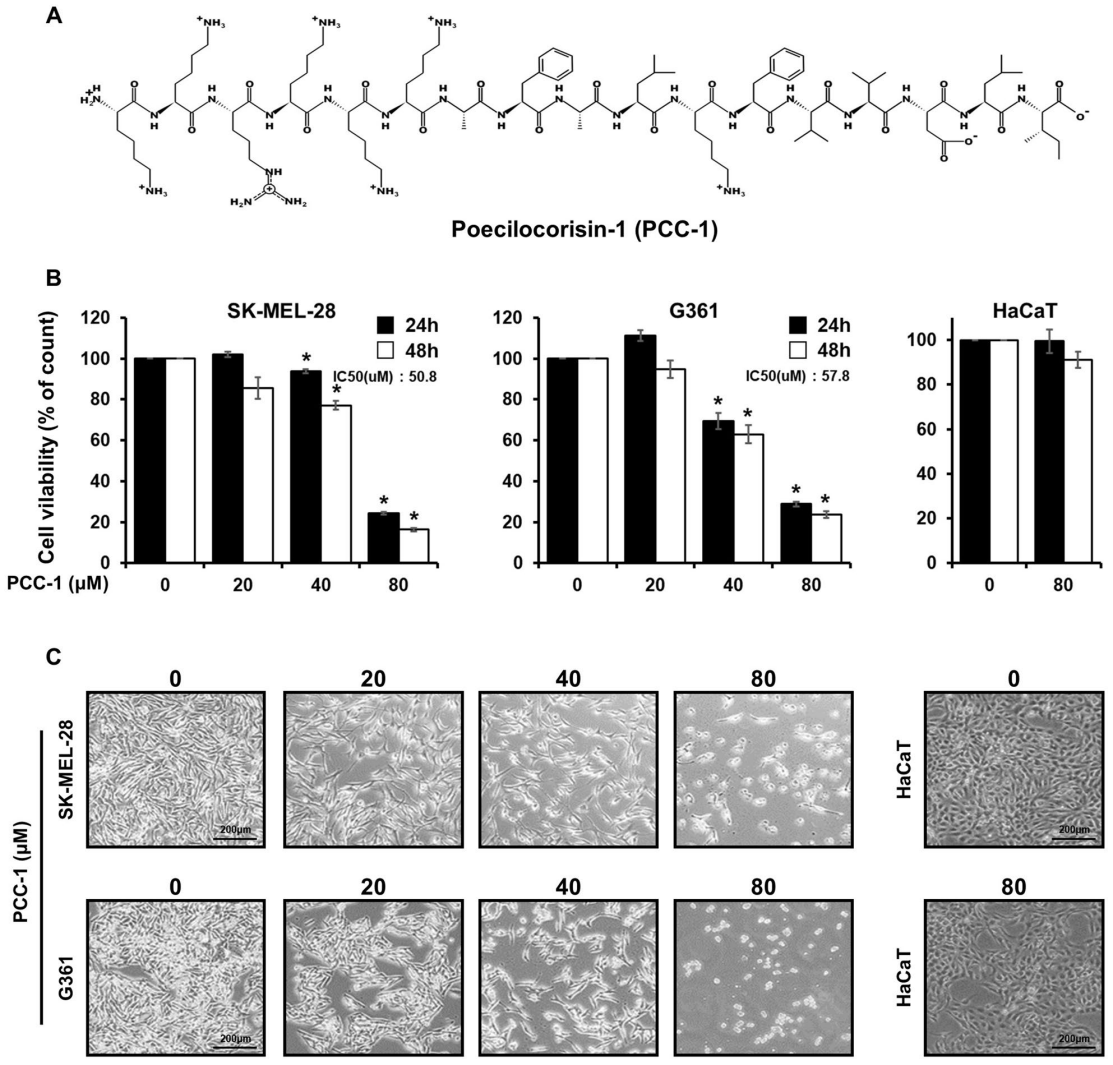
(**A**) Peptide structure of poecilocorisin-1 (PCC-1). (**B**) The cell viability in PCC-1-treated SK-MEL-28, G361, and HaCaT cells (20, 40, and 80 μM) was detected using an MTS assay kit. Data represent the mean ± standard deviation (SD). *Significantly different, as compared with untreated controls, by the paired *t* test (n = 3; p < 0.05). (**C**) Morphological changes observed in the PCC-1-treated SK-MEL-28, G361, and HaCaT cells (20, 40, and 80 μM) and untreated cells.

To analyze the cancer cell-specific cell viability of the peptide PCC-1 selected through the transcriptome analysis of *Poecilocoris lewisi*, SK-MEL-28, G361, and HaCaT cells were treated with various concentrations of PCC-1. The MTS assay was used to compare cell viability of untreated (control) skin cancer cells and normal cells at two time points (24 and 48 h), as shown in Fig. 1A,B. Both skin cancer cell lines showed decreased viability with increasing treatment concentration, which did not change significantly between the two time points (24 and 48 h). However, the decrease became significant as the treatment concentration increased over time. In addition, the IC50 values of SK-MEL-28 and G361 cells were 50.8 and 57.8 μM, respectively. However, the HaCaT cell line did not show a significant change when exposed to 80 μM PCC-1, showing the greatest decrease in cell viability. These results were verified by analyzing the morphological changes (Fig. 1C). SK-MEL-28 and G361 cell lines were treated with PCC-1 at 20, 40, and 80 μM for 48 h. The number of cells decreased, and the cells became more rounded. In contrast, HaCaT cells showed no change in morphology when PCC-1 was treated at 80 μM for 48 h.

### PCC-1 increases apoptosis in malignant melanoma cells

**Figure 2:**
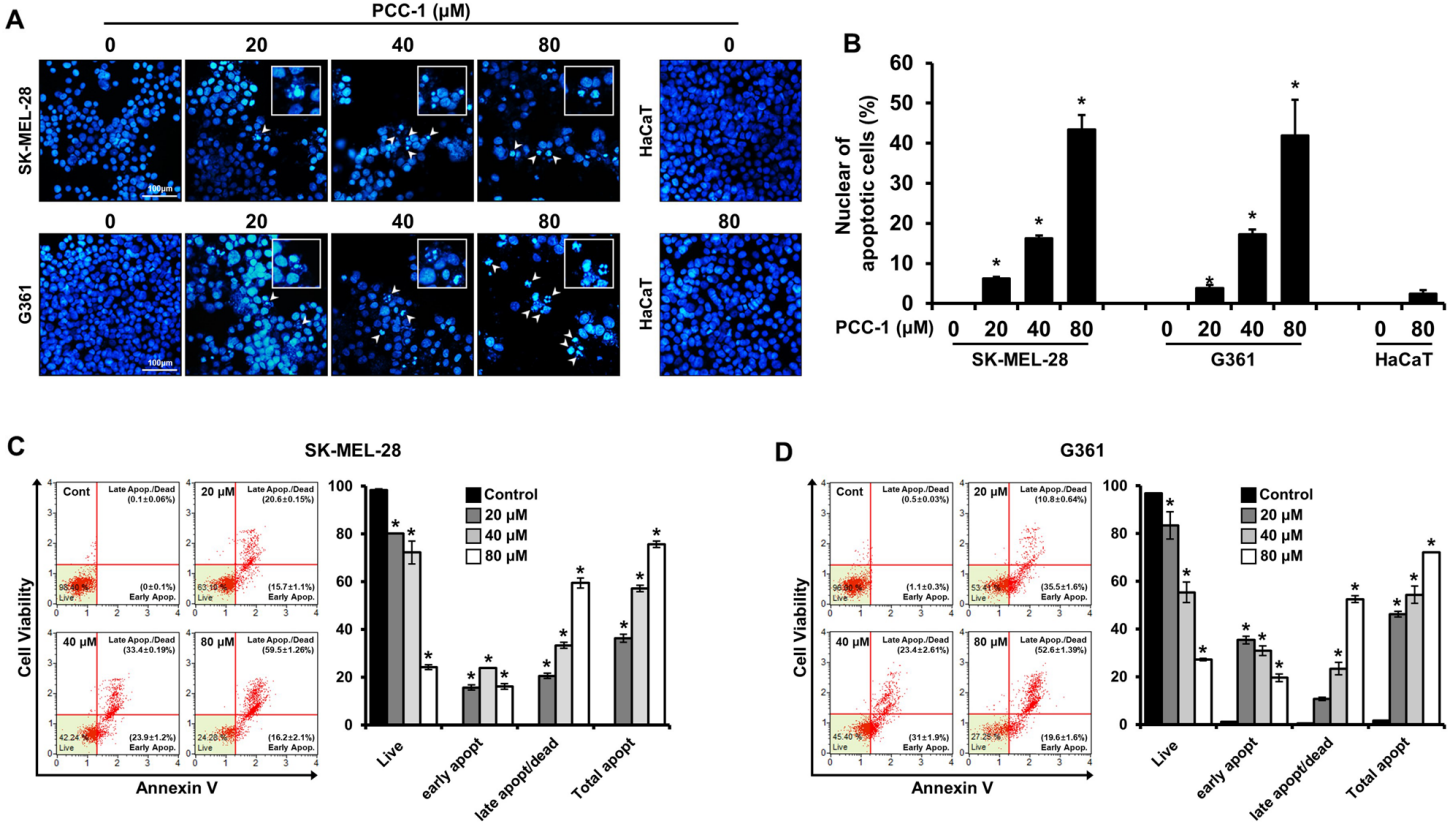
(**A**) Fluorescence microscopy images of DAPI-stained cells observed in poecilocorisin-1 (PCC-1)-treated SK-MEL-28, G361 (20, 40, and 80 μM) and HaCaT cells (80 μM). White arrows indicate DNA fragmentation and nuclear condensation. Scale bar = 100 μm. (**B**) DNA fragmentation and nuclear condensation were quantified, and data are presented as the mean ± standard deviation (SD) (n = 3; *p < 0.05). (**C**) SK-MEL-28 and (**D**) G361: Quantitative detection of Annexin V-positive cells using MuseTM Cell Analyzer. SK-MEL-28 and G361 cells were treated with PCC-1, and apoptosis was analyzed via Annexin V staining. Data represent mean percentage levels ± SD (n = 3; *p < 0.05).

In SK-MEL-28 and G361 cell lines, the apoptosis effect was increased by PCC-1 treatment, and this result was verified in the image of morphological changes. It was confirmed that apoptosis was induced by PCC-1 in SK-MEL-28 and G361 cell lines using DAPI staining analysis, which specifically stains the cell nucleus. Compared to the control nuclei, the cells cultured for 48 h after treatment with PCC-1 showed fragmented and condensed nuclei as the concentration of PCC-1 increased (20, 40, and 80 μM) On the other hand, morphological and nuclear changes were not observed in HaCaT cell lines when PCC-1 was treated at a concentration of 80 μM for 48 h (Fig. 2A,B). In addition, the apoptotic effect of PCC-1 on SK-MEL-28 and G361 cells was analyzed after 48 h using Annexin V staining (Fig. 2C,D). In Fig. 2C, the average percentage of early apoptotic cells in the SK-MEL-28 group was 0 ± 0.1, 15.7 ± 1.1, 23.9 ± 1.2, and 16.2 ± 2.1%, respectively, as the concentration of PCC-1 increased, and the ratio of dead cells and late apoptotic cells was, on average, 0.1 ± 0.06, 20.6 ± 0.15, 33.4 ± 0.19, and 59.5 ± 1.26%, respectively. The proportion of live cells in the control group was 98.4%, which decreased to 63.1, 42.24, and 24.28% as the treatment concentration increased. Figure 2D shows the proportions of the G361 group. The average percentage of cells in the initial apoptosis phase was 1.1 ± 0.3, 35.5 ± 1.6, 31 ± 1.9, 19.6 ± 1.6%, respectively, as the concentration of PCC-1 increased. The average percentage of dead cells and late apoptotic cells was 0.5 ± 0.03, 10.8 ± 0.64, 23.4 ± 2.61, and 52.6 ± 1.39% as the treatment concentration increased. The proportion of live cells in the control group was 96.9%, which decreased to 53.41, 45.4, and 27.25% as the treatment concentration increased. As the treatment concentration increased, the total apoptotic cell population increased, including dead, early and late apoptosis.

### PCC-1 regulates Sp1 protein levels in melanoma cells

**Figure 3:**
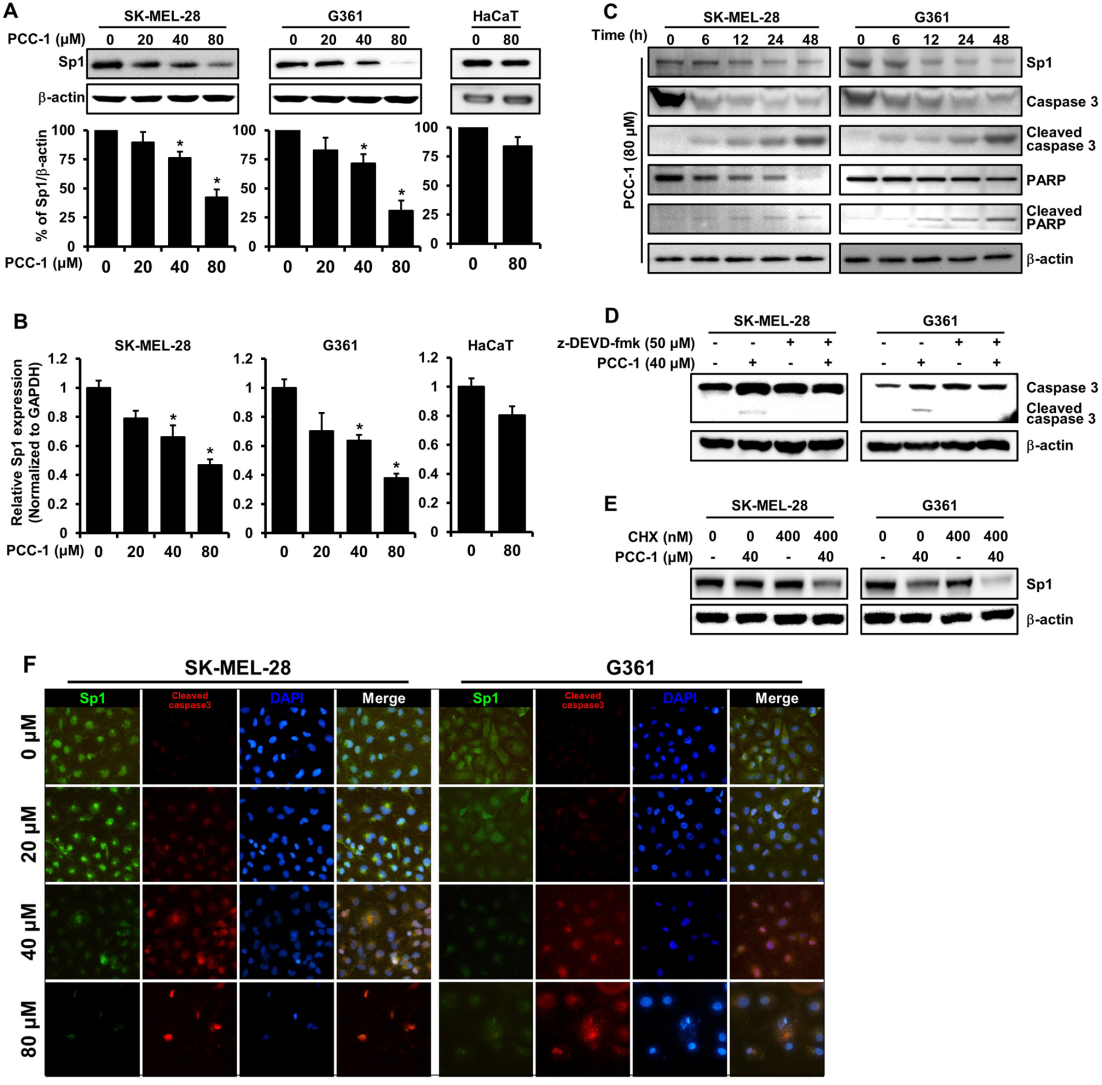
(**A**) SK-MEL-28, G361 and HaCaT cells were treated with poecilocorisin-1 (PCC-1) at 20, 40, and 80 μM for 48 h, and whole-cell extracts were prepared and separated on SDS-PAGE, followed by western blotting analysis for Sp1 antibodies. β-actin was used as a loading control. The graph represents the ratio of Sp1 to β-actin expression. (**B**) SK-MEL-28, G361 and HaCaT cells were treated with PCC-1 at 20, 40, and 80 μM for 48 h, and the levels of Sp1 mRNA normalized to those of GAPDH were measured by real-time PCR. (**C**) The dose- and time-dependent effects of PCC-1 on Sp1, caspase 3, cleaved caspase 3, PARP, and cleaved PARP were analyzed in SK-MEL-28 and G361 cells treated with PCC-1 (80 μM) at 6, 12, 24, and 48 h. Actin was employed as a loading control. (**D**) Analysis of the effect of PCC-1 on the apoptosis pathway following caspase3 inhibitor treatment. (**E**) Analysis of the intrinsic or extrinsic pathway of PCC-1 after translation inhibition following CHX treatment in skin cancer cell lines. Western blot analysis data complied with the digital image and integrity policy, and blots exposed from the same gel were used. (**F**) Immunofluorescence microscopy of SK-MEL-28 and G361 cells treated with PCC-1 at 20, 40, and 80 μM for 48 h. Cells were immunostained with anti-Sp1 and anti-cleaved caspase3, and signals were detected with with 488-conjugated and 647-conjugated secondary antibodies. DAPI was used for nuclear counterstaining. Data are expressed as the mean ± standard deviation (SD) of three independent experiments. Scale bar = 100 μm.

The transcription factor Sp1 has been studied that it is involved in cell cycle progression and apoptotic cell death^32–35^. The level of reduction in the expression of Sp1 according to the treatment concentration of PCC-1 analyzed through Western blot was evident. As the treatment dose of PCC-1 increased, the level of Sp1 protein (related to β-actin levels) decreased in both SK-MEL-28 and G361 cell lines. On the other hand, in the HaCaT cell line, when PCC-1 was treated at a concentration of 80 μM for 48 h, the protein level of Sp1 did not change (Fig. 3A, Supplementary Fig. 1). The comparison in the same amount of protein was ensured through a constant β-actin level, in particular the level of Sp1 expression decreased significantly when the treatment concentration of PCC-1 was 80 μM. In addition, it was confirmed through real-time PCR analysis that the level of Sp1 mRNA was decreased compared to the level of GAPDH mRNA. On the other hand, in the HaCaT cell line, when PCC-1 was treated at a concentration of 80 μM for 48 h, the mRNA level of Sp1 did not change (Fig. 3B). Consequently, in both Western blot and real-time PCR analysis, the levels of Sp1 protein and genes decreased as the treatment concentration of PCC-1 increased. Furthermore, it was confirmed that various apoptosis-related proteins such as caspase 3, PARP, and each truncated form, including Sp1, were time-dependently regulated when the concentration of PCC-1 was 80 μM (Fig. 3C, Supplementary Fig. 1). In addition, in order to analyze in more detail the involvement of PCC-1 in apoptosis, two cancer cells were treated with z-DEVD-fmk, which inhibits caspase3, and PCC-1. As a result, it was confirmed that cleaved caspase3 was not expressed after caspase3 was inhibited (Fig. 3D, Supplementary Fig. 1). We also inhibited translation using cycloheximide (CHX) to analyze the pathway by which PCC-1 induces apoptosis in skin cancer cell lines. As a result, it was confirmed that PCC-1 was involved in extrinsic apoptosis pathway through Sp1 protein regulation after translation was inhibited (Fig. 3E, Supplementary Fig. 1). Based on the above results, immunocytochemical analysis in SK-MEL-28 and G361 cell lines confirmed a decrease in the Sp1 level and an increased level of cleaved caspase3 in a concentration-dependent manner of PCC-1 (Fig. 3F). This study concludes that PCC-1 treatment in skin cancer cells leads to apoptosis through inhibition of Sp1.

### PCC-1 regulates cell cycle arrest and migration in melanoma cells

**Figure 4:**
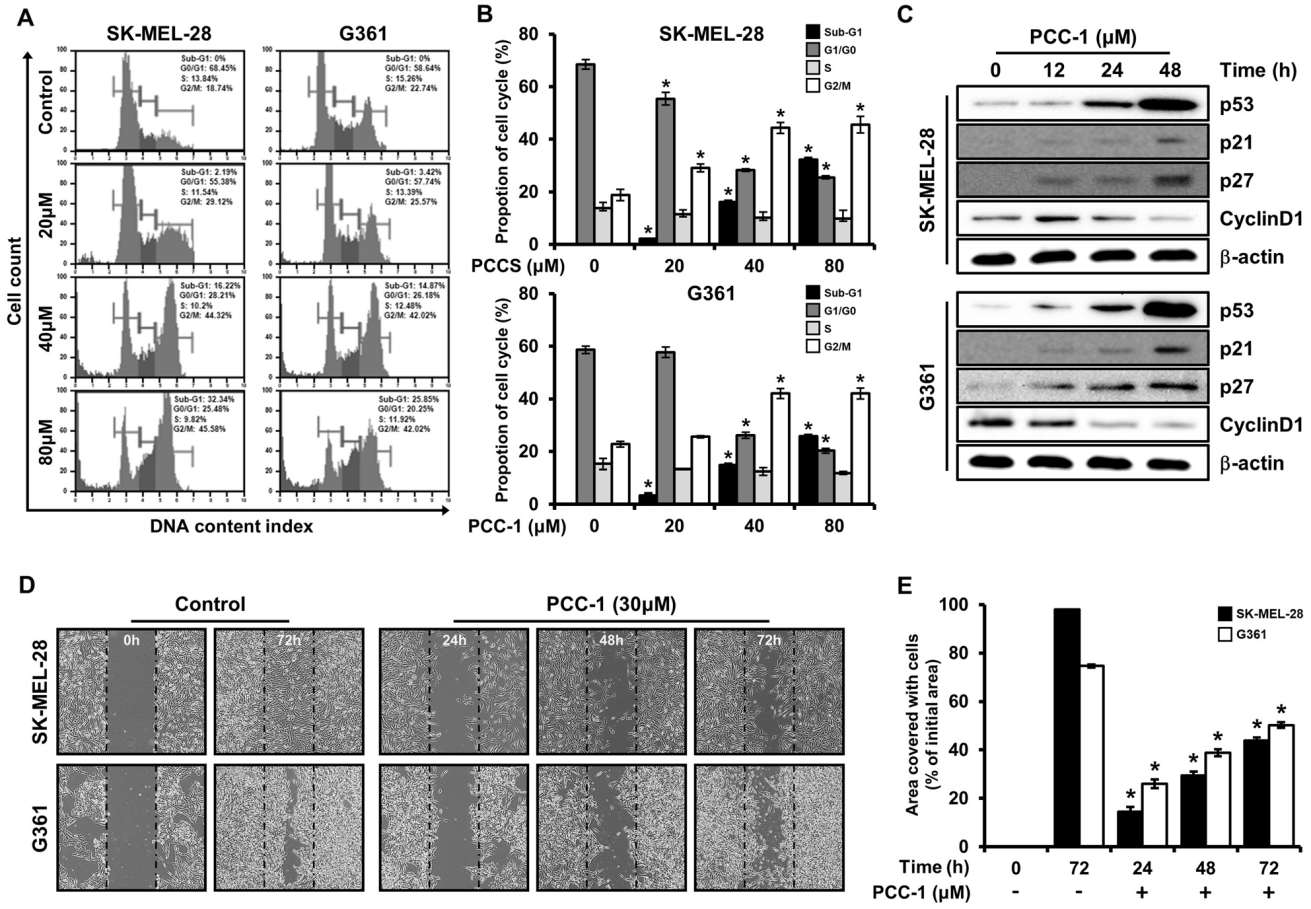
(**A**) SK-MEL-28 and G361 cells were treated with poecilocorisin-1 (PCC-1) (20, 40, and 80 μM) or untreated (control cells), and the cells were washed, fixed, stained with PI, and analyzed for DNA content by FACS analyzer. The percentage of apoptotic cells was measured using the Muse™ Cell Analyzer after propidium iodide (PI) staining. (**B**) The results of flow cytometry were quantified. Data represent the mean ± standard deviation (SD) (n = 3; *p < 0.05). (**C**) SK-MEL-28 and G361 cells were treated with PCC-1 (80 μM) and examined at 12, 24, and 48 h, and whole-cell extracts were prepared, separated on sodium dodecyl sulfate-polyacrylamide gel electrophoresis (SDS-PAGE), and subjected to western blotting using p53, p21, p27, and cyclin D1 antibodies. Actin was employed as a loading control. The results represent three independent experiments. Western blot analysis data complied with the digital image and integrity policy, and blots exposed from the same gel were used. (**D**) SK-MEL-28 and G361 cells were treated with PCC-1 (30 μM), and confluent cells were carefully scratched using sterile pipette tips and then re-cultured with or without PCC-1. At 24, 48, and 72 h, the cells were photographed under a microscope. (**E**) The results of the migration assay were quantified. Data represent the mean ± SD (n = 3; *p < 0.05).

In SK-MEL-28 and G361 cell lines, an analysis was performed on how the treatment of PCC-1 affects the cell cycle. During cell proliferation, G1/G0, S, and G2/M each represent different steps. Through analysis of the DNA content of control cells and PCC-1 treated (20, 40, and 80 μM) cells for 24 h after PCC-1 treatment, it was possible to determine the minimum time required for sufficient cell replication (Fig. 4A,B). Two typical G1/G0 and S peaks were observed in the control cells of both cell lines (Fig. 4A). As the treatment concentration of PCC-1 increased, a sub-G1 phase was appear indicating the formation of debris due to apoptosis. As the concentration of PCC-1 increased from 20 to 80 μM, the percentage of cells in the sub-G1 phase gradually increased with the change in the total percentage of cell cycling (such as G1/G0, S, and G2/M). In addition, when treated with 20 μM of PCC-1 compared to the control cells, the percentage of cells in the G1/G0 phase increased to 68.45% and 58.64%, respectively, for SK-MEL-28 and G361. This result means that PCC-1 is treated so that cells in the G1/G0 phase cannot proceed to the subsequent S phase, which induces cell cycle arrest. Then, the proteins p53, p21, p27, and cyclin D1 involved in advancing the cell cycle to the next phase in G1/G0 were analyzed (Fig. 4C, Supplementary Fig. 1). Expression of negative cell cycle regulatory proteins such as p53, p21 and p27 increased with time from 0 to 48 h in both cell lines, SK-MEL-28 and G361, respectively, after treatment with 80 μM PCC-1. In contrast, the levels of cyclin D1, a positive cell-cycle regulation protein, decreased as time elapsed. The time-dependent regulation of protein expression in cell cycle-related proteins indicates that cell cycle arrest and apoptosis are caused by PCC-1 treatment. Figure 4D shows the proliferation rate of cell migration when PCC-1 was treated with 30 μM, which is similar to that observed in the in vitro metastasis. The two control cell lines SK-MEL-28 and G361 almost recovered 72 h after scratching the center of the monolayer, but the PCC-1 treated group did not recover for the same time. In contrast, the group treated with PCC-1 increased by about 20% after 24 h, and the recovery area gradually increased after 48 h and 72 h, but compared with the control group, the group treated with PCC-1 for 72 h Only about 45% of the rate was recovered (Fig. 4D,E). Based on these results, it was confirmed that when PCC-1 was treated with SK-MEL-28 and G361 cell lines, a certain range of proliferation was inhibited, it can be strongly assumed that this result is directly related to the expression of the various proteins analyzed above.

## Discussion

Peptides have various therapeutic effects, and among them, they have been attracting attention for their excellent safety and anticancer efficacy. In particular, discovering therapeutic peptides for the development of anticancer drugs is a novel and promising approach^8,9^. ACPs are divided into antimicrobial/pore-forming peptides, cell-permeable peptides, and tumor-targeting peptides groups according to their main mechanisms of action^28^. AMPs (antimicrobial/pore-forming peptides) has specific biological activity and occurs naturally in all living organisms^10^. They have the potential as antimicrobial agents such as defensins and cathelicidins, and are part of an innate immune defense mechanism^36,37^. These AMPs form an amphiphilic structure in a non-polar solvent, and most of them are short and have a cationic charge^38^. They disrupt function through electrostatic interactions that bind to negatively charged bacterial cell membranes, leading to cell death^38,39^. Pore-forming peptides that induce cell death through such necrosis or apoptosis target cancer cell membranes. In necrosis, AMP destroys the mitochondrial membrane to induce apoptosis and causes cell lysis by targeting negatively charged molecules in the cancer cell membrane^28^. One of the AMPs with these properties, the insect-derived peptide CopA3, inhibits the growth of pancreatic and liver cancer cells and has antibacterial activity^16,29^. This study is a representative study of synthetic peptides based on bioactive substances occurring in nature with potential as novel anticancer agents of AMPs that exhibit inhibitory activity against microorganisms.

Based on these findings, a peptide with new anticancer potential was selected during the analysis of the transcriptome of *Poecilocoris lewisi* in a previous study^30^. PCC-1 is a peptide derived from *Poecilocoris lewisi*, which has excellent antibacterial and antifungal effects. Moreover, PCC-1 inhibits NO secretion and the expression of IL-6, iNOS, and COX-2Therefore, PCC-1 has potential as a therapeutic agent for various inflammatory diseases (Fig. 1A)^50^. As the first step to elucidate the anticancer efficacy of PCC-1, melanoma cells (SK-MEL-28 and G361 cell lines) were treated with the peptide, and the cytotoxic and cancer cell proliferation inhibitory effects were confirmed at this time. However, the human epithelial cell line HaCaT did not show toxicity, and through MTS analysis, it was confirmed that PCC-1 has anticancer efficacy specifically for skin cancer cells (Fig. 1B,C).

Sp1 represents an essential promoter in cancer cells and plays an important role as a basal transcription factor. Numerous studies on Sp1 in various cancer cells have been reported^3,31,40–43^. In addition, Sp1 expression levels are higher in cancer cells than in normal cells, and the growth and metastasis of cancer cells are weakened in nude mice in which Sp1 is knocked out^44,45^. Therefore, lowering Sp1 levels is a good strategy to prevent tumor cell growth. However, the anticancer effects of Sp1 using the insect-derived peptide PCC-1 have not been reported yet. To the best of our knowledge, this is the first study to demonstrate the anticancer effects of PCC-1.

To analyze the anticancer effect of PCC-1 on the melanoma cell lines, the results of the untreated cells and cells treated with various concentrations of PCC-1 were compared. Furthermore, Sp1 protein is closely related to apoptosis and cell cycle, and changes in the expression levels of related regulatory proteins were observed. For this, DAPI staining and Annexin V analyses were performed to classify the different stages of apoptosis based on cell morphology (early apoptosis, late apoptosis, and dead cells). As the concentration of PCC-1 increased, abnormal DNA fragmentation was observed with DAPI staining in both SK-MEL-28 and G361 cell lines (Fig. 2A,B). In addition, total apoptosis increased, and the percentage of living cells decreased with increasing treatment concentrations (Fig. 2C,D). These apoptotic effects were analyzed by western blotting and real-time PCR analysis (Fig. 3A,B), and the expression levels of Sp1, caspase 3, PARP, cleaved caspase 3, and cleaved PARP were consistent with and provided detailed insights into the cell viability test results (Fig. 3C,F). Additionally, we inhibited caspase3 through z-DEVD-fmk to determine how PCC-1 affects the apoptosis pathway. In skin cancer cell lines, cleaved caspase3 was not expressed when PCC-1 was treated in a state where caspase3 was inhibited, and from this result, it was confirmed that PCC-1 was involved in the apoptosis pathway of cancer cells (Fig. 3D). In addition, in order to check which pathway (intrinsic or extrinsic) PCC-1 regulates Sp1 in skin cancer cell lines, translation was inhibited through CHX and then PCC-1 was treated. As a result, it was confirmed that PCC-1 is involved in the extrinsic apoptosis pathway through the regulation of Sp1 protein after translation is inhibited. However, considering that PCC-1 regulates the mRNA level of Sp1, it is presumed that PCC-1 acts directly or indirectly in the intrinsic or extrinsic pathway (Fig. 3E).

Based on the result that increasing PCC-1 concentration induced apoptosis in melanoma cells, the expression of Sp1 target proteins, including p53, p21, p27, and cyclin D1^46^, were investigated in terms of cell cycle. Cell cycle arrest was analyzed based on measurements of DNA content at specific stages of the cell cycle, and the number of cells in the sub-G1 phase increased with increasing dosing time (Fig.4A,B). The p53, p21, and p27 proteins are negative regulators of the cell cycle^47,48^, and the kinase of the protein is upregulated, resulting in cell cycle arrest that blocks progression to the next phase (Fig. 4C). In contrast, cyclin D1, which is involved in tumor formation, is a positive modulator^49^. Thus, positive and negative modulators exhibited cell cycle arrest and increased the duration of the lower G1 phase. Lastly, through wound healing analysis, a direct wound was caused to the cancer cells, and when PCC-1 was administered to the melanoma cells, the recovery ability of the cancer cells was suppressed compared to the control group (Fig. 4D,E).

In conclusion, our study demonstrated that the insect-derived peptide PCC-1 induces cell proliferation inhibition, apoptosis, and cell cycle arrest by downregulating Sp1 expression in the melanoma cell lines SK-MEL-28 and G361. The results of this study support the anticancer efficacy of PCC-1, which regulates Sp1 in human cancer cells, for clinical application. Furthermore, we are currently researching on the possibility of a safe and effective peptide that can be applied clinically by studying the interaction between the peptide and an immune checkpoint inhibitor based on the results of this study.

## Materials and methods

### Peptide synthesis

The homodimeric peptide PCC-1 (KKRKKKAFALKFVVDLI-NH_2_) was synthesized using the solid-phase peptide synthesis method by GL Biochem Ltd. (Shanghai, China). The peptide was dissolved in acidified distilled water (0.01% acetic acid) and stored at 20 °C until use. Peptide synthesis was performed as previously described^16,29^.

### Cell lines and culture conditions

The malignant melanoma cell lines SK-MEL-28 (KCLB No. 30072) and G361 (KCLB No. 21424) were obtained from the Korean Cell Line Bank (KCLB, Seoul, Korea). A non-tumoral immortalized human epidermal cell line,HaCaT, was provided from Mokpo University (Muan-gun). Cells were grown routinely in Dulbecco’s Modified Eagle’s Medium (DMEM; Biowest, Pays De La Loire, France) with 10% fetal bovine serum (FBS) and 100 U/mL each of penicillin and streptomycin (Gibco, Grand Island, NY, USA) in appropriate concentrations at 37 °C with 5% CO_2_ in a fully humidified atmosphere. Cell culture was performed as previously described^50^.

### MTS cell viability assay

The effect of PCC-1 on cell viability was estimated using a MTS (3-(4,5-dimethylthiazol-2-yl)-5-(3-carboxymethoxyphenyl)-2-(4-sulfophenyl)-2H-tetrazolium) assay kit (Promega, Madison, WI, USA). SK-MEL-28 (2.5 × 10^3^), G361 (3 × 10^3^) and HaCaT (2.5 × 10^3^) cells were seeded in 96-well plates, respectively, and PCC-1 was treated at each concentration (0, 20, 40, and 80 µM) for 24 h and 48 h. After treating each well with the MTS solution, the cells were incubated for 3 h. Absorbance was measured at 490 nm was recorded using a GloMax-Multi + Microplate Multimode Reader (Promega). The viability of PCC-1-treated cells was represented as a percentage after being normalized to those of untreated control cells. MTS assay was performed as previously described^50^.

### DAPI staining

Nuclear condensation and fragmentation were observed by nucleic acid staining with DAPI. SK-MEL-28 and G361 cells treated with PCC-1 were harvested by trypsinization and fixed in 100% methanol at room temperature (RT) for 20 min. The cells were seeded onto slides, stained with DAPI (2 μg/mL), and monitored using an inverted fluorescence microscope (Korea Lab Tech, KI-3000F, Gyeonggi-do, Korea). DAPI staining analysis was performed as previously described^50^.

### Annexin V staining

SK-MEL-28 (3 × 10^5^ cells/well) and G361 (3 × 10^5^ cells/well) cells were seeded into six-well plates and cultured overnight. After cells were treated with different concentrations (20, 40, and 80 µM) of PCC-1 for 48 h, they were stained with Muse™ Annexin V and Dead Cell reagents (Muse™ Apoptosis Assay kit, EMD Millipore). Stained cells were analyzed using the Muse™ Cell Analyzer. Annexin V staining analysis was performed as previously described^50^.

### Cell-cycle analysis

Cells were seeded into six-well plates and exposed to PCC-1 at different concentrations (20, 40, and 80 µM) for 24 h. Cells were harvested and washed three times with phosphate-buffered saline (PBS). The harvested cells were fixed in 70% ethanol at 20 °C overnight. Cells were washed with PBS, mixed with 200 µL of Muse™ cell-cycle reagent (EMD Millipore, Billerica, MA, USA), and incubated at RT for 30 min in the dark. Cell cycle was analyzed by flow cytometry using the Muse™ Cell Analyzer (EMD Millipore, Billerica, MA, USA). Cell-cycle analysis was performed as previously described^50^.

### Immunocytochemical testing

SK-MEL-28 and G361 cells were seeded onto each sterilized glass coverslip on six-well tissue culture plates for 24 h, treated with PCC-1 (20, 40, and 80 µM), and then incubated for 48 h. The cells were fixed/permeabilized with cytotoxic solution (BD Biosciences, San Jose, CA, USA) for 20 min at 4 °C. The cells were incubated with anti-Sp1 and anti-cleaved caspase3 in 0.5% BSA at 4 °C overnight. After each cell was washed with PBST, Sp1 antibody was incubated with the Alexa Fluor 488-conjugated anti-mouse, cleaved caspase3 antibody was incubated with 647-comjugated anti-rabbit secondary antibodies (Jackson ImmunoResearch, West Grove, PA, USA), respectively, and DAPI was stained in the dark for 30 min at RT. The cells were visualized under an inverted fluorescence microscope (Korea Lab Tech, KI-3000F, Gyeonggi-do, Korea). Immunocytochemical analysis was performed as previously described^50^.

### Western blot analysis

SK-MEL-28 and G361 cells were treated with PCC-1 (20, 40, and 80 µM) and then incubated for 48 h, washed with PBS, and then lysed with RIPA Mammalian Protein Extraction Reagent (Thermo Scientific, Rockford, IL, USA). Extracted proteins were quantified using the Pierce BCA Protein Assay Kit (Thermo Scientific). Equal amounts of the protein samples were separated by 10% or 15% sodium dodecyl sulfate-polyacrylamide gel electrophoresis (SDS-PAGE) and then transferred to membranes. The membranes were blocked for 1 h at RT with 5% non-fat dried milk in PBS containing 0.1% Tween-20 and then incubated overnight at 4 °C with specific antibodies. Protein bands were observed after treating the membranes with horseradish peroxidase-conjugated secondary antibodies using a Pierce ECL Western Blotting Substrate (Thermo Scientific). Western blot analysis was performed as previously described^50,51^.

### Wound healing assay

A scratch wound healing assay was performed as previously described^52,53^. Briefly, SK-MEL-28 and G361 cells were grown in 100 mm culture dishes. The cell monolayer was scratched with a sterile pipette tip and washed with PBS to remove cell debris. Then, PCC-1 was treated with 30 μM and cultured for 24, 48 and 72 h to observe cell proliferation under a microscope. Wound healing assay was performed as previously described^50^.

### Statistical analysis

Results were presented as means ± standard deviation (SD) of triplicate independent experiments. Statistical significance was assessed using a Student’s *t* test. A value p < 0.05, as compared with the non-treated control, was considered statistically significant. Statistical analysis was performed as previously described^50^.

## Supplementary Information

Supplementary Figure 1.
