# Supplementary material for: Antitumorigenic effect of insect-derived peptide poecilocorisin-1 in human skin cancer cells through regulation of Sp1 transcription factor
Source: Sci Rep. 2021 Sep 16;11:18445. doi: 10.1038/s41598-021-97581-0 (PMC8446052; doi:10.1038/s41598-021-97581-0)
Supplement: Supplementary file 1 — Supplementary Figure 1. [file 41598_2021_97581_MOESM1_ESM.docx]

**Supplementary material**

**Western blot original**

**Figure 3A**

| **Sp1** |  |
| --- | --- |
| **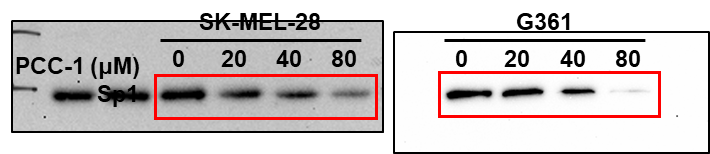** | **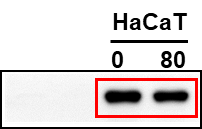** |
| **β-actin** |  |
| **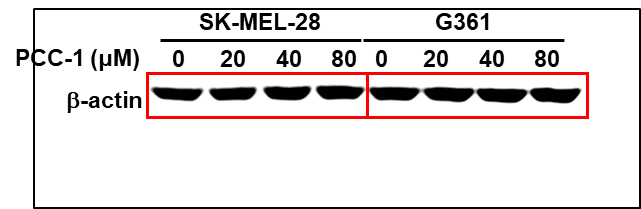** | **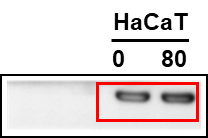** |

**Figure 3C**

| **Sp1** |
| --- |
| **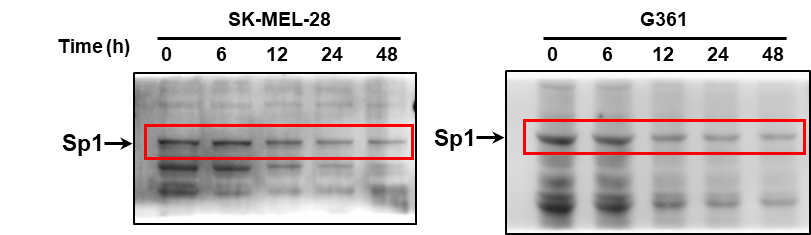** |

| **Caspase3** |
| --- |
| **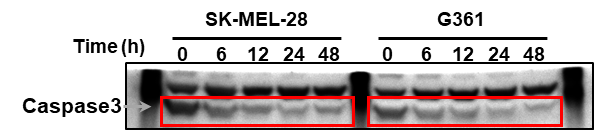** |

| **Cleaved caspase3** |
| --- |
| **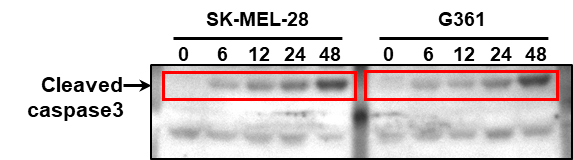** |

| **PARP & Cleaved PARP** |
| --- |
| **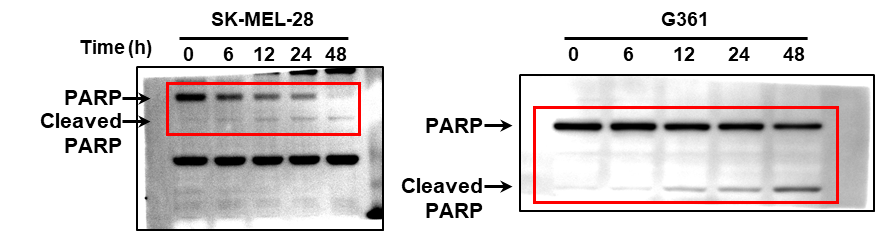** |

| **β-actin** |
| --- |
| **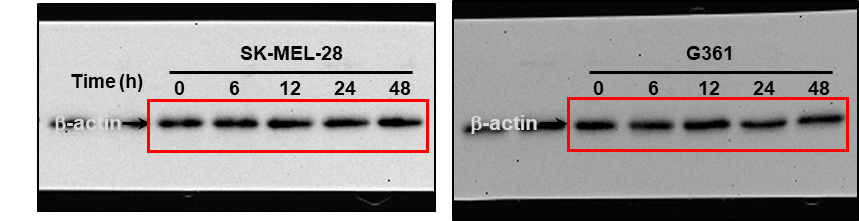** |

**Figure 3D**

| **Caspase3 & Cleaved caspase3** |
| --- |
| **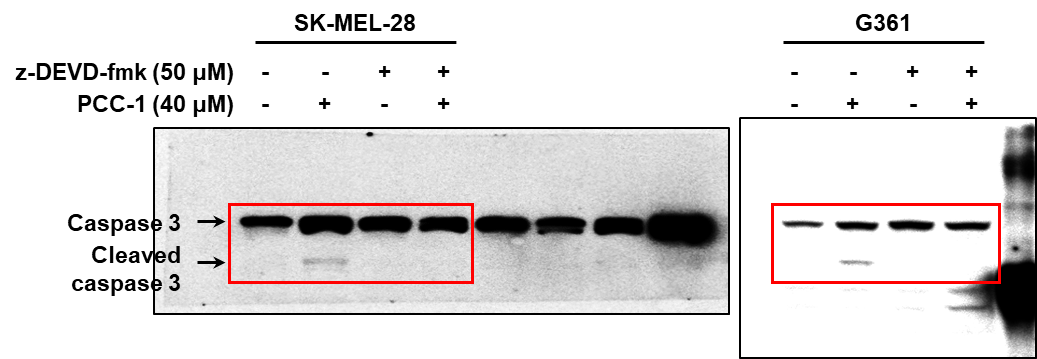** |

| **β-actin** |
| --- |
| **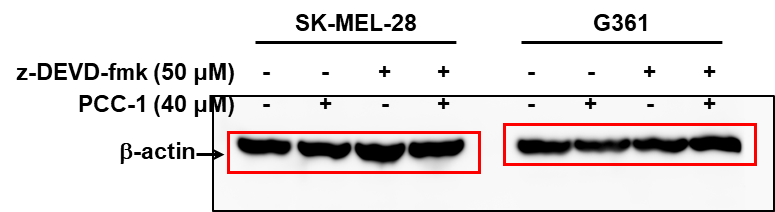** |

**Figure 3E**

| **Sp1** |
| --- |
| **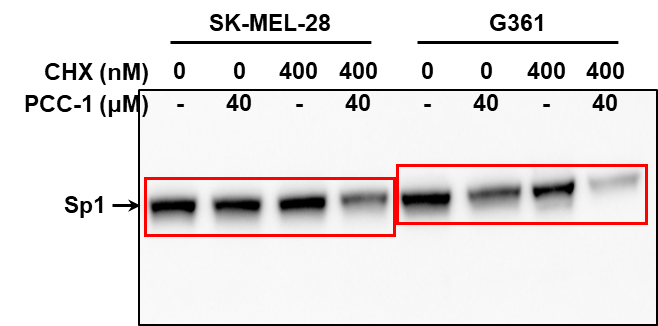** |

| **β-actin** |
| --- |
| **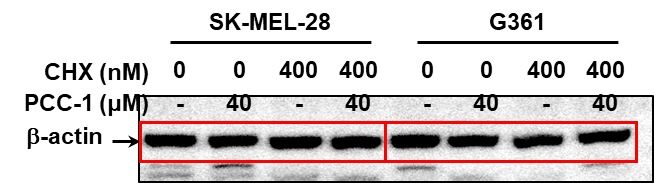** |

**Figure 4A**

| **p53** |
| --- |
| **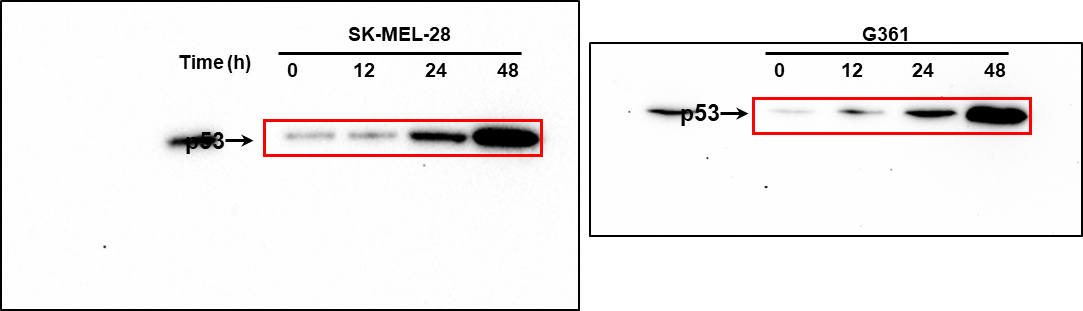** |

| **p21** |
| --- |
| **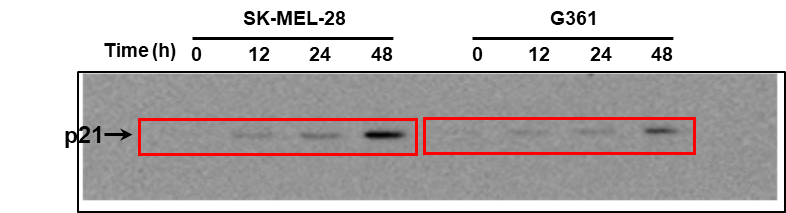** |

| **p27** |
| --- |
| **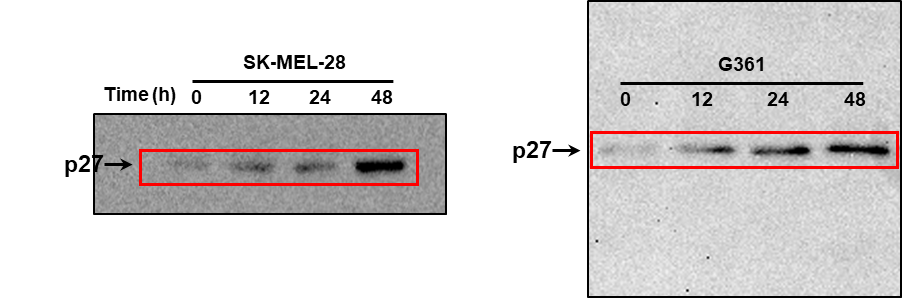** |

| **CyclinD1** |
| --- |
| **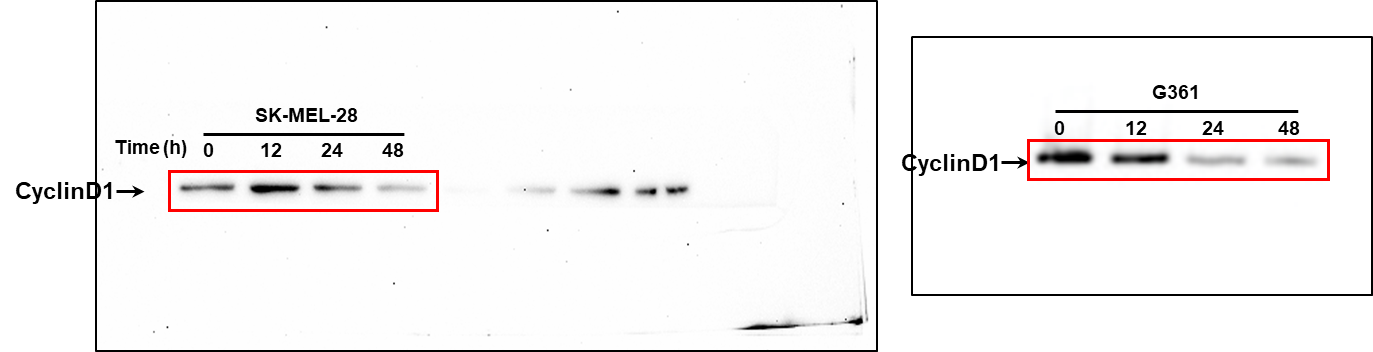** |

| **β-actin** |
| --- |
| **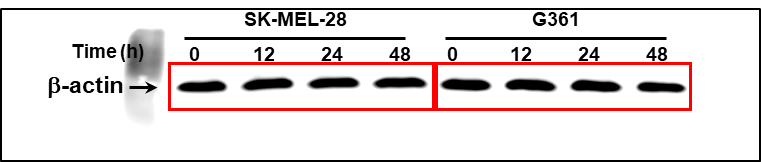** |
